# Skeletal Muscle Metastasis of a GIST: A Case Report and Review of the Literature

**DOI:** 10.1155/2016/7867545

**Published:** 2016-12-25

**Authors:** Olga D. Savvidou, George D. Chloros, Georgios D. Agrogiannis, Penelope Korkolopoulou, Georgios N. Panagopoulos, Panayiotis J. Papagelopoulos

**Affiliations:** ^1^The First Department of Orthopaedic Surgery, “Attikon” Hospital, University of Athens School of Medicine, Athens, Greece; ^2^First Department of Pathology, Laikon General Hospital, School of Medicine, Athens University, Athens, Greece

## Abstract

Gastrointestinal stromal tumors (GISTs) are the most common malignant mesenchymal tumors of the gastrointestinal tract. The most common sites of metastasis are the liver and the peritoneum, whereas metastasis to soft tissue is rare. The authors present the case of a 78-year-old male with a soft tissue metastasis of a GIST and the current literature is reviewed.

## 1. Introduction

Gastrointestinal stromal tumors (GISTs) are the most common sarcomas occurring in the gastrointestinal (GI) tract [1]. They are considered to be rare neoplasms, representing only 0.1%–3% of all GI malignancies [2, 3]. However, they account for 80% of GI mesenchymal neoplasms [1]. They usually involve the stomach, grow variably, present vaguely with inconsistent clinical behavior ranging from relatively benign to highly aggressive behavior [4, 5]. Pathogenesis involves a gain-of-function mutation in a KIT (tyrosine-protein kinase Kit) and 95% of GIST cases are immunohistochemically positive for the receptor tyrosine kinase KIT, also known as CD117 [6]. GIST remains an unpredictable entity, with almost 50% of patients developing recurrence or metastasis within 2 years of presentation [7, 8]. Anatomic site, tumor size, and mitotic activity are among the most popular factors proposed to predict GIST behavior [9, 10]. Advanced disease usually presents with liver lesions and peritoneal seeding [9]. Alternative metastatic sites such as skeletal muscle are exceedingly rare [11–16]. We hereby present a case of metastasis to the adductor magnus muscle of a 78-year-old male previously diagnosed with GIST of the rectum. The patient provided informed consent for publication of this case report.

## 2. Case Report

**Figure 1:**
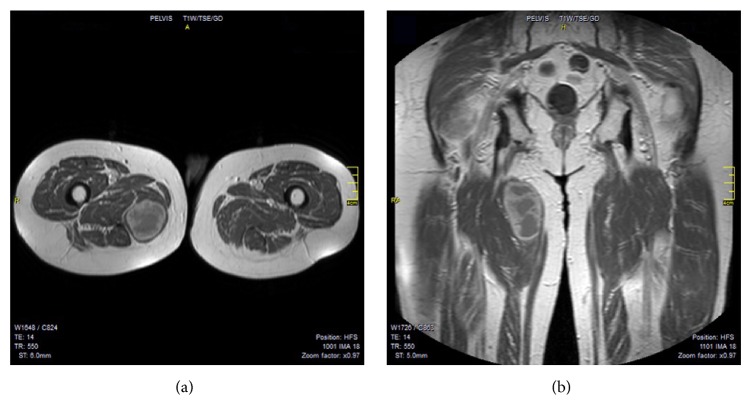
Axial (a) and coronal (b) T1 views of an MRI of the lesion in the context of the right adductor magnus muscle, with sparse areas of necrosis and irregular contrast enhancement.

A 78-year-old male presented to the authors' institution after noting a painful soft tissue mass located in his medial right upper thigh 4 months previously. Eight years priorly, he was hospitalized for lower GI bleeding. A colonoscopy performed at that time revealed the presence of a soft and friable rectal mass 2 cm proximal to the anal sphincter. He subsequently underwent transanal endoscopic sphincter-sparing surgery. Histology revealed the presence of a GIST. The patient remained free of disease for 3 years, when routine surveillance revealed local recurrence. The patient underwent interval transanal surgical resection and was started on systemic imatinib mesylate, which was discontinued 2 years later after the development of considerable side effects of nausea and vomiting. The patient was disease-free with no evidence of recurrence or metastatic disease until his current visit at the authors' institution due to the aforementioned painful soft tissue mass. An MRI with contrast enhancement of the right thigh was obtained, including T1- and T2-weighted as well as fat suppression images, which revealed the presence of a 9.0 × 6.0 × 5.0 cm lesion in the right adductor magnus muscle, with sparse areas of necrosis and irregular contrast enhancement (Figure 1). Bone scanning Tc99m showed an area of increased metabolic activity in the right thigh with no other abnormalities indicative of metastases.

**Figure 2:**
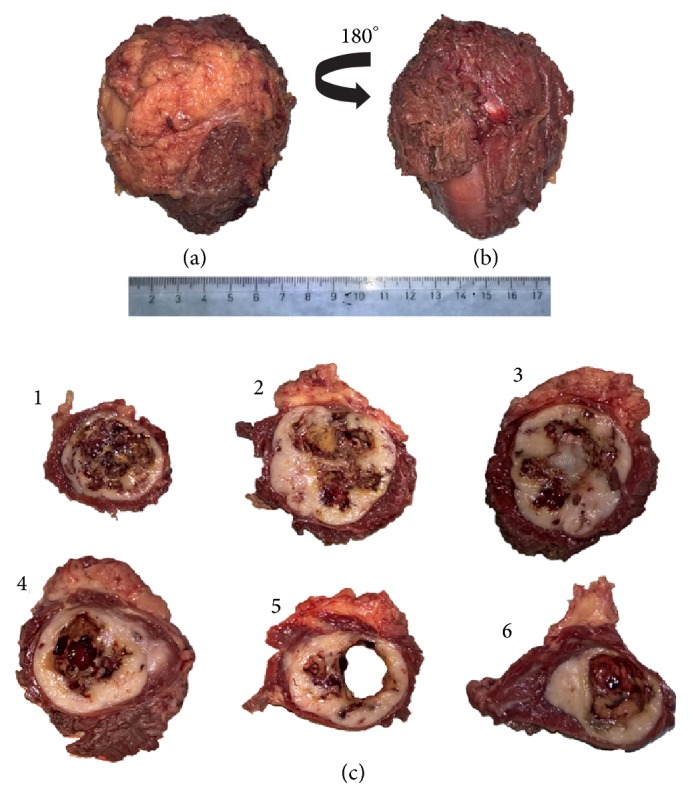
Gross appearance of the resected specimen. (a) Superficial view; (b) deep view; (c) sectioning of the mass, showing central necrosis and macrocystic change (1–6: proximal to distal).

**Figure 3:**
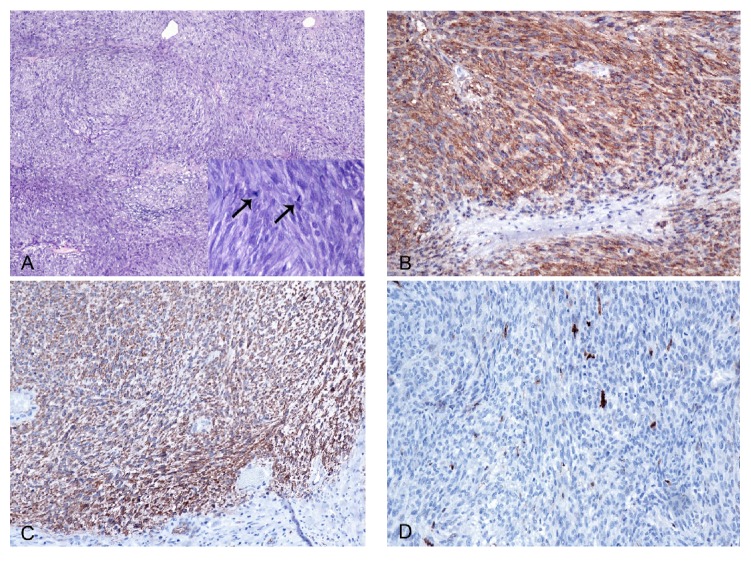
Cellular neoplasm, arranged in fascicles and whorls, with high mitotic rate (arrows indicate mitotic figures). (A) Hematoxylin-eosin stain. (B), (C), and (D) immunohistochemical staining for c-KIT, CD34, and S100, respectively. All images were captured under 200x original magnification.

A percutaneous CT-guided biopsy was performed which was consistent with a tumor of mesenchymal origin. The patient subsequently underwent wide excision of the lesion under general anesthesia. Grossly, the tumor presented as an intramuscular mass of 9 cm in largest diameter. Upon sectioning, it showed central necrosis and macrocystic change (Figure 2). Histopathological examination revealed a hypercellular neoplasm. The neoplastic cells were arranged in either a fascicular or a whirling pattern and exhibited spindle and epithelioid morphology (Figure 3(A)). The mitotic index was high, counting over 10 mitoses per 20 high power fields. Areas of hemorrhage, myxoid change, and necrosis were also identified. At the periphery, the tumor was focally infiltrating the surrounding muscle fibers. Based on these findings, the tumor was classified in the prognostic group 6a (high grade) based on the Miettinen and Lasota/AFIP criteria for metastatic risk [17], which is in concordance with the malignant behavior of the tumor. Immunohistochemically, the tumor cells were strongly positive for c-KIT and CD34, focally positive for SMA, and negative for desmin and S100 (Figure 3(B, C, and D)). Interestingly, both the metastatic and primary tumors were negative for DOG-1. These findings were consistent with metastatic infiltration of the adductor magnus by a GIST. Surgical margins were free of disease for at least 5 mm. Instructions were given for close oncologic surveillance thereafter. At the last follow-up, 18 months postoperatively, the patient demonstrated no signs of local or systemic disease.

## 3. Discussion

GISTs are rare neoplasms, representing only 0.1%–3% of all GI malignancies [2, 3]. However, they account for 80% of GI mesenchymal neoplasms [1]. Approximately 4500–6000 GISTs are diagnosed annually in the United States [18, 19]. Median age at diagnosis is 60 years with no sex, racial, or ethnic predilection [11]. They may originate anywhere in the GI tract but the most common location is the stomach (60%), followed by the jejunum and ileum (30%), the duodenum (5%), and the colon and rectum (<5%) [4, 5]. The typical gross morphology of GIST is a soft, friable, and highly vascular tumor which often predisposes to bleeding. It may cause life-threatening hemorrhage because of protrusion from the site of origin and/or by eroding the bowel lumen [1]. Other common symptoms at presentation, which are not pathognomonic as they are not solely related to GISTs, include abdominal pain, nausea, vomiting, anemia, and weight loss [18, 20]. Thus, diagnosis of GIST may be challenging due to their frequently vague presentation [6].

Among the modalities used during the initial workup of diagnosing a GIST, CT with contrast, endoscopic ultrasound (EUS) and EUS-guided fine needle aspiration (EUS-FNA) are the most popular [1]. Moreover, positive immunohistochemical markers for c-KIT, CD34, and reverse transcriptase polymerase chain reaction (rt-PCR) analysis for KIT mutations are usually necessary to confirm the diagnosis and differentiate them from other tumors [1]. Differentiating skeletal muscle metastases from GIST and primary soft tissue sarcomas, such as primary leiomyosarcomas, may be challenging due to their almost identical cell morphology, as they both appear with cellular bundles of spindle cells [16]. In a study of 133 GISTs, 97.75 (130 cases) percent had been initially misdiagnosed as either leiomyosarcoma, smooth muscle tumors of uncertain malignant potential, or leiomyoma and only 2.25 (3 cases) percent had been correctly diagnosed as GISTs [21]. Thus, a thorough histopathologic workup, including the use of a basic panel of immunohistochemical stains, is critical, in order to differentiate the aforementioned tumors, as their prognostic and therapeutic implications are quite different. In the case presented herein, c-KIT and CD34 were positive, therefore posing the correct diagnosis.

Due to their resemblance under light microscopy, GISTs were not regarded as a distinct pathological entity in earlier literature but as smooth muscle neoplasms and most were designated as leiomyomas, leiomyoblastomas, leiomyosarcomas, or schwannomas. GISTs are now thought to ascend from the interstitial cells of Cajal or their stem cell precursors, which are normally part of the autonomic nervous system of the gastrointestinal tract and operate as a pacemaker in controlling GI motility [22, 23]. This finding was followed by the development of therapeutic agents that suppress tumor growth by specifically inhibiting this signal (imatinib mesylate, sunitinib malate) [1, 24]. Complete surgical resection with no tumor rupture remains the gold standard of treatment along with adjuvant TKI therapy, typically consisting of imatinib mesylate [25]. Our patient had initially received imatinib which had to be discontinued due to significant side effects. Between 15% and 50% of patients with GIST have already metastatic disease at diagnosis. Common sites of metastasis include the liver, peritoneum, and omentum [26]; metastases to lymph nodes and extra-abdominal structures (lung, bone, soft tissue, and brain) are uncommon (<5%) [11]. Soft tissue metastasis of a primary GIST in skeletal muscle is exceptionally rare. To our knowledge, only 5 cases have been reported in English literature [12–16]. Pasku et al. [15] reported a case of bilateral gluteal and lung metastases in a 56-year-old female with a GIST of the pelvis, initially misdiagnosed as a uterine leiomyosarcoma. She was treated with total resection of the tumor and adjuvant imatinib. Bashir et al. [12] presented the case of a 56-year-old male with upper back muscle, adrenal gland, and cardiac metastases of a small intestine GIST. The tumor was excised and the patient received TKI therapy. Final outcome was not reported. Suzuki et al. [16] reported the case of a 54-year-old male, presenting with an enlarging mass of the left buttock, initially misdiagnosed as leiomyosarcoma. After surgical excision and immunohistochemical study of the tumor the patient was ultimately diagnosed with a GIST of the small intestine and skeletal muscle metastasis. Despite treatment, the patient died of overt GI bleeding 6 months after diagnosis. Cichowitz et al. [13] reported the case of a 23-year-old female with a 6 cm adductor longus muscle lesion, presenting 5 years after initial resection of an 11 cm small bowel GIST. She underwent resection of the lesion, which confirmed the diagnosis of metastatic GIST to muscle. Finally, Jin et al. [14] reported the case of an 80-year-old female, presenting with a soft tissue mass in the setting of a gastric GIST with omental invasion, diagnosed 3 years before. An incisional biopsy confirmed the presence of a solitary metastatic muscle mass. Due to the patient's unstable health state the lesion was not excised and she received TKI therapy.

There are a few sparse hypotheses regarding the potential factors that might contribute to the development of skeletal muscle metastasis of a GIST. Unfavorable mechanical and metabolic environment in skeletal muscle seems to trigger neoplastic cell death. On the contrary, injured or denervated muscle might be more vulnerable to metastatic seeding [27].

In our case, an unusual finding was represented by the fact that the resected mass was immunohistochemical DOG-1 (a novel GIST marker) negative. There is evidence in current literature suggesting that imatinib therapy might lead to the development of atypical tumor clones, which are often negative for common GIST markers, such as CD117, CD34, and DOG-1 [28, 29]. While it remains uncertain if this development leads to a more aggressive clinical behavior, it may cause diagnostic confusion. Awareness of this misleading phenomenon can prevent false interpretation of the lesion as “non-GIST” [29]. Musculoskeletal or other metastases in GIST represent a hallmark of advanced disease.

In conclusion, even though skeletal muscle metastases from GISTs are rare, the likelihood of identifying metastases in unusual sites is increasing due to major improvement in the progression-free survival and overall survival rates of patients with GISTs. Additionally, side effects from TKIs are common, and therefore skeletal muscle metastases should likely be totally surgically excised.
